# Testes-specific protease 50 (TSP50) promotes invasion and metastasis by inducing EMT in gastric cancer

**DOI:** 10.1186/s12885-018-4000-y

**Published:** 2018-01-23

**Authors:** Qing-Hua Cao, Fang Liu, Chang-Zhao Li, Ni Liu, Man Shu, Yuan Lin, Li Ding, Ling Xue

**Affiliations:** 1 Department of Pathology, The First Affiliated Hospital of Sun Yat-sen University, #58, Zhongnshan Road II, Guangzhou, 510080 China; 2 Department of Oncology, Nanfang Hospital of Southern Medical University, Guangzhou, China; 3 Department of Dermatology and Skin Diseases Research Center, University of Alabama at Birmingham, Alabama, USA

**Keywords:** TSP50, Proliferation, Invasion, EMT, Gastric cancer

## Abstract

**Background:**

TSP50 (testes-specific protease 50) has been reported to be a candidate oncogene and is overexpressed in various cancers. Our previous study demonstrated that TSP50 protein is elevated in gastric cancer, and its high expression is associated with unfavorable prognosis and lymph node metastasis. However, the role of TSP50 in gastric cancer remains elusive.

**Methods:**

qRT-PCR, western blot were used to determine TSP50 expression in gastric cancer cell lines. Role of TSP50 in proliferation and invasion was examined by BrdU incorporation assay, cell count, wound healing and transwell assay. Immunohistochemistry and western blot were performed to identify the potential mechanisms involved.

**Results:**

TSP50 was highly expressed in most of the gastric cancer cell lines at both mRNA and protein levels. Up-regulation of TSP50 in gastric cancer cells enhanced proliferation and invasiveness, whereas down-regulation of TSP50 by its specific shRNA decreased it. A negative correlation between TSP50 and E-Cadherin was found in gastric cancer tissues, and combination of them improves the prediction for prognosis and lymph node metastasis. Mechanistic studies revealed that overexpression of TSP50 increased the expression of epithelial-to-mesenchymal transition (EMT) markers including Vimentin, and Twist, and decreased the epithelial marker E-Cadherin. NF-κB signaling pathway is involved in the regulatory effects of TSP50 on EMT, migration and invasion in gastric cancer cells.

**Conclusion:**

TSP50 promotes the proliferation, migration and invasion of gastric cancer cells involving NF-κB dependent EMT activation. Targeting TSP50 may provide a novel therapeutic strategy for the management of gastric cancer.

**Electronic supplementary material:**

The online version of this article (10.1186/s12885-018-4000-y) contains supplementary material, which is available to authorized users.

## Background

Gastric cancer is the second leading cause of cancer death in China, accounting for about 17.6% of all cancer deaths [1], although its incidence and mortality rates decreased worldwide [2]. Despite advances in diagnosis and treatment, the outcome of gastric cancer patients remains poor [3, 4]. Local invasion and distal metastasis largely account for the poor prognosis in these patients. Therefore, it is imperative to explore the underlying mechanism of gastric cancer metastasis in order to identify novel therapeutic approaches and to improve the patient survival.

Testes-specific protease 50 (TSP50) is a novel member of cancer/testis antigens (CTAs), which is not expressed in normal tissues except testes and cancers, including breast cancer, colorectal cancer, laryngocarcinoma and cervical cancer [5–10]. Accumulating evidences implicate that TSP50 is involved in proliferation, apoptosis, migration and metastasis in various types of tumors except gastric cancer [8–10]. We recently reported that TSP50 expression was up-regulated in gastric cancer tissues compared with adjacent non-tumor mucosa. TSP50 overexpression was associated with lymph node metastasis and poor prognosis in gastric cancer patients [11]. However, the biologic role and molecular mechanisms of TSP50 in gastric cancer metastasis remain to be elucidated.

NF-κB is known to be a tumorigenic and prometastatic factor in gastric cancer [12, 13]. NF-κB signaling has been proved to be a downstream target of TSP50 [14, 15]. Moreover, deregulation of NF-κB has been reported to induce the epithelial-to–mesechymal transition (EMT) in various cancers [16–18], which is believed to be an essential step for tumor cell invasion and metastasis.

In this study, we investigated whether TSP50 activates EMT in gastric cancer cells by NF-κB signaling pathway thus promoting cancer invasion and metastasis. Genetic manipulation of TSP50 levels showed that TSP50, being highly expressed in gastric cancer cells, promoted the proliferation, migration and invasion in vitro. Mechanistically, TSP50 activates EMT in gastric cancer by up-regulating Vimentin and Twist whereas down-regulating E-Cadherin. The control of TSP50 on EMT activation was also confirmed in human gastric cancer tissues. Statistical analysis showed a significant negative correlation between TSP50 and E-Cadherin expression in human gastric cancer tissues. Combining TSP50 and E-Cadherin provide superior performance in the prediction of prognosis and metastasis as compared to TSP50 or E-Cadherin alone. In addition, we showed that TSP50 activates EMT process in gastric cancer via augmenting NF-κB signaling pathway. Pharmacological inhibition of NF-κB pathway by its specific inhibitor blocks TSP50 induced migration and invasion. Our data for the first time identified the mechanism by which TSP50 may promote tumor cell invasion and metastasis in gastric cancer.

## Methods

### Patients and specimens

Formalin-fixed, paraffin-embedded tissues from 334 patients with gastric cancer were collected as described in our previous study [11], 30 corresponding lymph node metastatic lesions were added in the present study.

### Cell lines and cell culture

Human gastric adenocarcinoma cell line MKN-45, BGC-823, MGC-803, SGC-7901, AGS and human gastric epithelial cell line GES-1 (Shanghai Institute of Cell Biology, China) were grown in F-12 k (ATCC) supplemented with 10% fetal bovine serum and 1% penicillin-streptomycin at 37 °C with humidified 5% CO_2_. For inhibitor treatment, the cultured cells were incubated with 10 μmol/l BAY-117082 (Selleck, USA) for 48 h. Cells were collected in logarithmic growth phase for all experiments.

### RNA extract and quantitative real time PCR

Total RNA was extracted from MKN-45, BGC-823, MGC-803, SGC-7901, AGS and GES-1 using Trizol reagent (Invitrogen, USA) according to protocol. Complementary DNA was prepared using oligodT primers according to the protocol supplied with the Primer Script TM RT Reagent (TaKaRa, Japan). Expression of TSP50 was determined by quantitative real-time PCR using Power SYBR green PCR master mix (Applied Biosystems). Results were normalized to the expression of GAPDH. The primers for TSP50 were: forward: 5’-TCGTGCTCGTTCCAAAGG-3′ and reverse: 5’-GGCAATAGGTGGGTTCGTT-3′.

### Establishment of stably transfected cell lines

For TSP50 overexpression, ectopic TSP50 coding sequence was amplified by polymerase chain reaction (PCR). The primer sequences were: forward: 5’-GTAGGATCCGCGAGGGGAAGCCCCGG-3′ and reverse: 5’-CCGAATTCTTATCACTGCCCGTTGAGGCAGTCC-3′. The amplified product was cloned into the pBaBb-puromycin plasmid and confirmed by sequencing. For TSP50 and p65 silencing, sequences of short hairpin RNA targeting TSP50 (shTSP50) and p65 (shp65) werecloned into the pSUPER-retro-puromycin plasmid. The shTSP50 and shp65 sequences were: 5’-GTTCTGCTATGAGCTAACT-3′ and 5’-GCCCTAUCCCTTTACGTCA-3′, respectively. The sequence of scrambled control shRNA was: 5’-GACGCTTACCGATTCAGAA-3′. GC cell lines were transfected with aforementioned constructed plasmids or empty vector. Stably transfected cell lines were selected with 0.5 μg/ml puromycin at 48 h after infection.

### Cell proliferation assay

BrdU incorporation and Cell count were used to assess cell proliferation as described previously [19]. BrdU incorporation was examined using 5-Bromo-2′-deoxy-uridine Labeling and Dectection kit III (Roche Applied Science, Mannheim, Germany) according to the manufacturer’s instructions. Briefly, cells were serum free for 24 h. Then cells were trypsinized and equal number (2 × 10^4^) of cells from each group was plated into a 96-well plate and grown in complete culture medium with 10 μM BrdU for 2, 4 or 6 h. BrdU incorporation into cellular DNA was assessed using a microplate reader (Safie II; Tecan, Mannedorf, Switzerland). The experiment was repeated three times independently. For the cell counts, cells were serum free for 24 h. Then cells were trypsinized and equal number (2 × 10^5^) of cells from each group was plated into 6-well culture plates in complete culture medium for 1, 2, 3 days. Then the cell number was determined in triplicate using a hemocytometer.

### Cell migration and invasion assay

Wound healing assay and transwell assay were employed to evaluate the ability of migration and invasion. For the wound healing assay, cells were serum free for 24 h. Then cells were trypsinized and equal number (3.5 × 10^5^) of cells from each group was plated into 6-well culture plates in complete culture medium for 4 h, then a scratch lesion was created using a 200 μl pipette tip. To avoid dislodged cells, culture medium was removed and wells were washed gently with PBS. Then cells were grown in serum-free culture medium for 24 h until the digital images of cells migrated into the scratch were taken on an inverted microscope. Measurement of wound area was done using the Adobe Photoshop software. Wound closure was quantified as the mean ± standard deviation(SD) of three independent experiments. The control wound closure was set as 100%, and the wound closure of overexpression or knockdown group was represented as the percent of the control. Transwell inserts with 8 μm pores (BD Biosciences, San Jose, CA, USA) for transwell migration assays, 2 × 10^5^ cells in serum free medium were added to each upper compartment of the chamber. After 48 h incubation, noninvasive cells were removed from the upper surface of the transwell membrane, and migrated cells were fixed with methanol, stained with 1% crystal violet and counted using a light microscope in 5 random visual fields at the magnification of 100 × .

### Immunohistochemistry analysis

Immunohistochemistry was carried out with the Dako Envision System (Dako, Denmark). Target protein expression level was evaluated by integrating the percentage of positive tumor cells and the intensity of positive staining. Briefly, sections were scored as 0 (negative), 1 (bordering), 2 (weak), 3 (moderate) or 4 (strong), whereas the staining extent was scored according to the area percentages: 0 (0%), 1 (1–25%), 2 (26–50%), 3 (51%–75%) or 4 (76–100%). The products of staining intensity and extent scores were the final staining scores (0–16). The median score was used as cut-off point to divide the patients into high or low expression group.

### Western blot analysis

Cells were collected and lysed with the RIPA buffer containing protease inhibitor. Protein concentration was determined by the Bradford method with bovine serum albumin as the control. Equal amounts of tissue lysates (50 μg) were run by SDS-PAGE, and electro-transferred on a polyvinylidene difloride membrane. The membrane was then blocked and incubated with primary antibodies against TSP50 (1:400, Proteintech, USA), E-cadherin (1:500, Abcam, UK), Vimentin(1:2000, Abcam, UK), Twist(1:1000, Abcam, UK), P65(1:500, Santa Cruz, CA), Histone1 (1:500, Dako, Denmark) and β-actin antibody (1:1000, Santa Cruz, CA) respectively, for 2 h at room temperature, and then incubated with appropriate horseradish peroxidaseconjugated secondary antibodies (1:1000, Cell Signaling Technology) for 1 h at room temperature. Final detection was carried out with LumiGLO chemiluminescent reagent (New England Biolabs) as described by the manufacturer. The densities of target bands was accurately determined by the computer-aided 1-D gel analysis system.

### Statistical analysis

Statistical analysis was performed using SPSS standard version 19.0 (SPSS Inc) and GraphPad Prism 5 (GraphPad Software). Survival curves calculation and OS curve plotting used the Kaplan-Meier method, and the Log-Rank test was applied to compare the distribution between patient subsets. The association between TSP50 and E-Cadherin was estimated by Phiand Cramers V correlation analysis. ANOVA or Student’s unpaired t-test were used to analyze the cellular proliferation, migration and invasion. Data from all quantitative assays were demonstrated as the mean ± standard and values of *P* < 0.05 were considered statistically significant.

## Results

### Elevated expression of TSP50 in gastric cancer cells

**Fig. 1:**
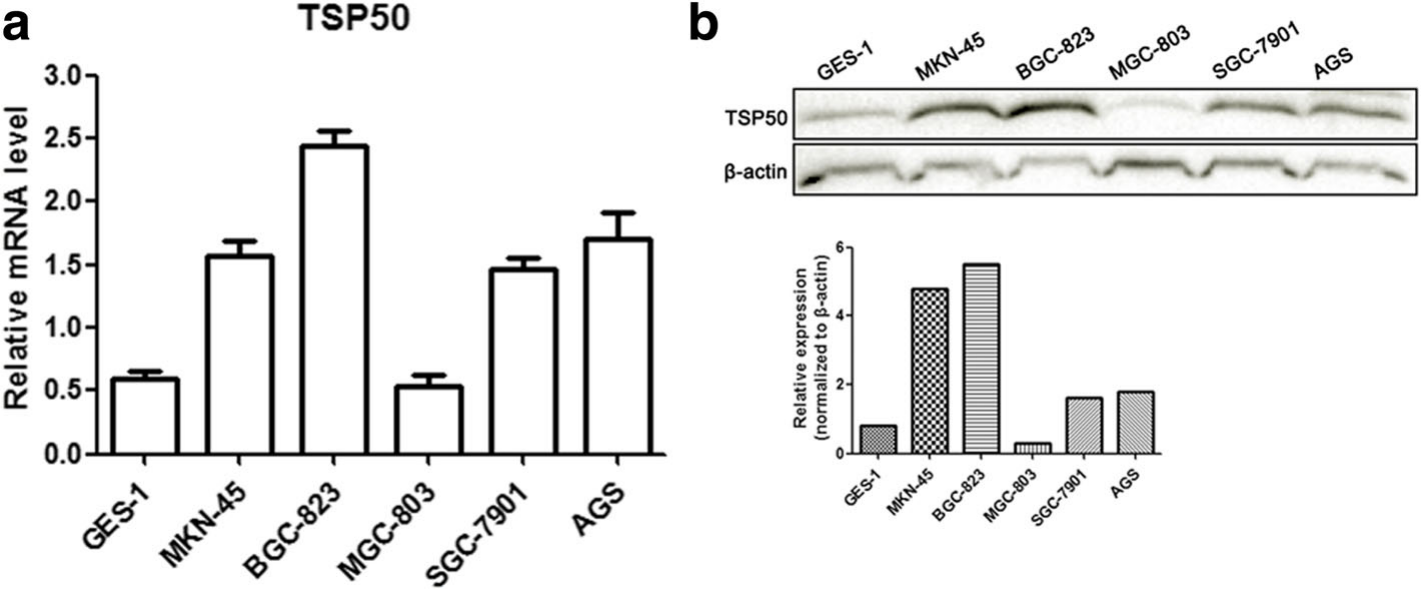
Elevated expression of TSP50 in gastric cancer cell at mRNA and protein level. **a** Relative expression level of TSP50 was evaluated by quantitative real time PCR in gastric cancer cells and human gastric epithelial cell line GES-1. **b**TSP50 protein was measured by western blot analysis in gastric cancer cells and GES-1. β-actin served as an inner control. Data are expressed as the mean ± SD from three independent experiments

Our previous study showed that TSP50 was overexpressed in gastric cancer tissues compared to adjacent non-tumor mucosal tissues [11]. To further investigate the biological function of TSP50 in gastric cancer, we first tested TSP50 expression levels employing several gastric cancer cell lines (MKN-45, BGC-823, MGC-803, SGC-7901, AGS) and GES-1 cell (normal human gastric epithelial cell). Quantitative PCR and western blot analysis revealed that the level of TSP50 expression was higher in almost all gastric cancer cell lines, except MGC-803, when compared to GES-1 cell at both the mRNA and protein levels (Fig. 1a, b). Particularly, its expression was found to be most up-regulated in BGC-823 cell lines. Therefore, we chose MGC-803 and BGC-823 cell lines to investigate the role of TSP50 in gastric cancer in overexpression and knockdown experiments respectively..

### Overexpression of TSP50 promotes proliferation, migration and invasion of MGC-803 cell

**Fig. 2:**
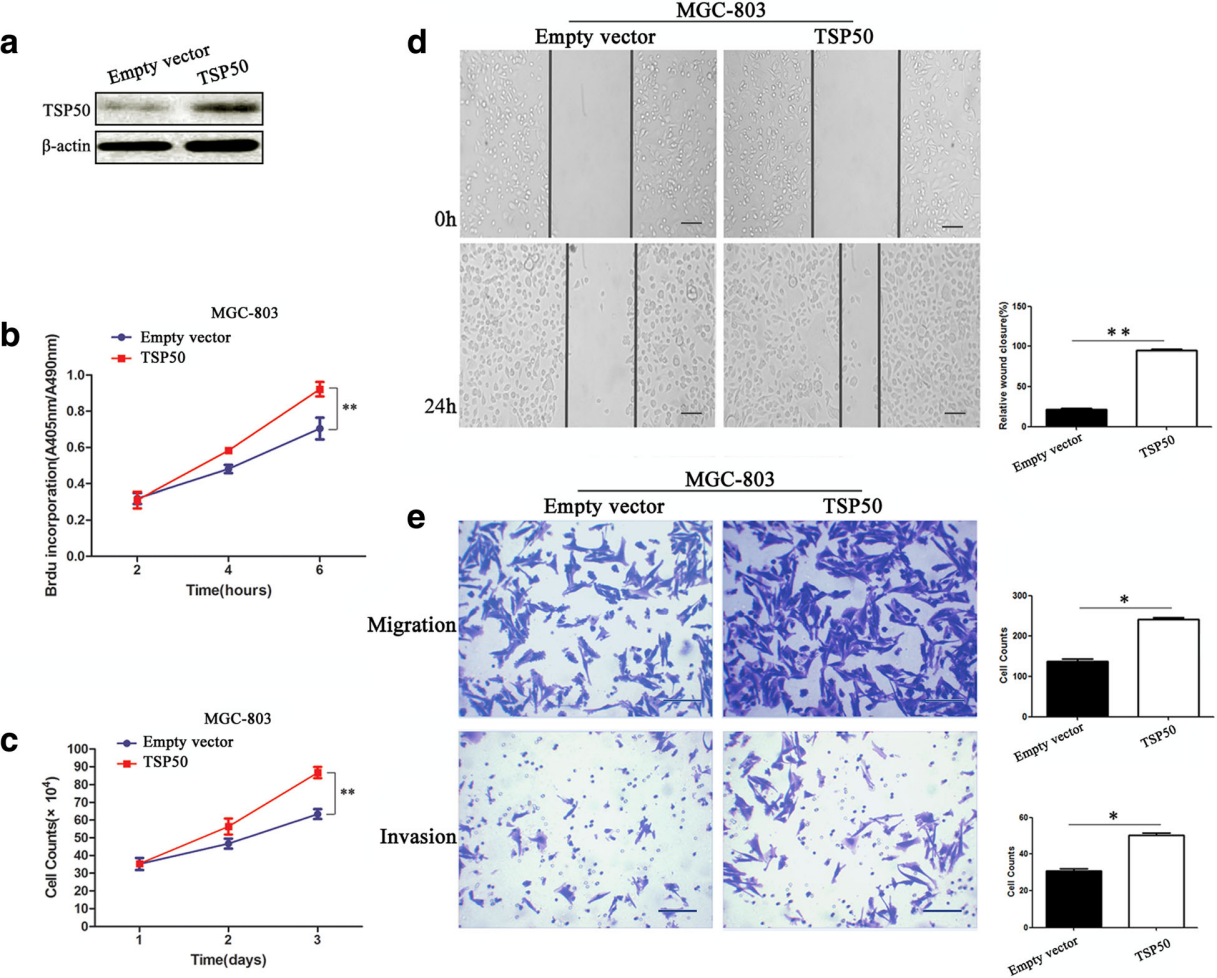
Overexpression of TSP50 promotes proliferation, migration and invasion in MGC-803 cell. **a** MGC-803 cell transfected with TSP50 overexpression plasmid. The efficiency of transfection was confirmed by western blot. β-actin served as an inner control. **b** BrdU assay showed a significant increase in BrdU uptake in MGC-803 cell transfected with TSP50 overexpression plasmid compared to that of the control group. (** *P* < 0.01). **c** Cell count experiment detected remarkable increase of cell number in overexpression-TSP50 MGC-803 cell compared to that of the control group. (** *P* < 0.01). **d** In wound closure assay, the migratory speed of MGC-803 cell transfected with TSP50 overexpression plasmid increased significantly compared to that of the vector control. (** *P* < 0.01). **e** In the transwell assay, migrated cells significantly increased after transfected with TSP50 overexpression plasmid. (* *P* < 0.05). In the transwell invasion assay, invasiveness was quantified by cells through Matigel and it was showed more cells in MGC-803 cell transfected with TSP50 overexpression plasmid than vector control. (* *P* < 0.05). All data are expressed as the mean ± SD from three independent experiments. Scale bar = 100 μm

In our previous study, TSP50 expression positively correlated with lymph node metastasis status and later disease stage [11]. Therefore, here we utilized genetic approaches to manipulate the TSP50 levels in gastric cancer cells thereby to explore its role in proliferation, migration and invasion in gastric cancer cells. First, ectopic overexpression of TSP50 was performed in MGC-803 cells which did not harbor higher TSP50 protein expression level than normal gastric epithelial cells (Fig. 2a). To show the effect of TSP50 on cellular proliferation in MGC-803 cells, we utilized BrdU incorporation assay. A significant increase of BrdU uptake in MGC-803 GC cells transfected with TSP50 expressing plasmid suggests that TSP50 promotes proliferation in these cells (Fig. 2b). This effect was further confirmed by cell count experiments, in which a significantly higher number of cells were recorded in TSP50 overexpressed cells (Fig. 2c). We further examined the effect of TSP50 on migration and invasion in MGC-803 cell. Results from wound healing assay suggested that overexpression of TSP50 in MGC-803 cell significantly enhanced its migratory speed compared to that of the vector control (Fig. 2d). Similarly, as shown in Fig. 2e, TSP50 overexpression significantly promotes the invasive ability of MGC-803 cell compared to that of the vector control in transwell assay.

### Knockdown of TSP50 inhibits proliferation, migration and invasion of BGC-823 cell

**Fig. 3:**
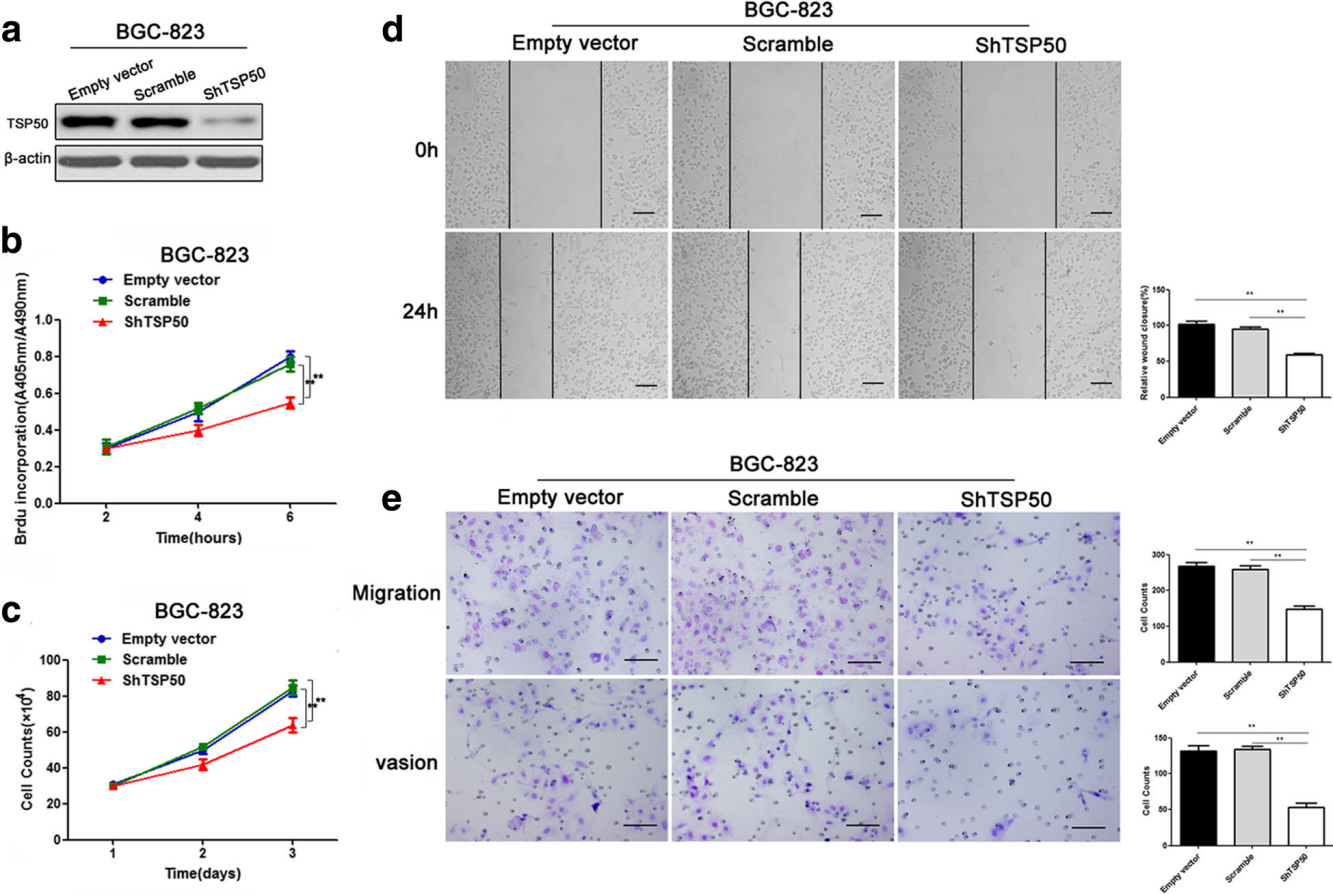
Knockdown of TSP50 inhibits proliferation, migration and invasion in BGC-823 cell. **a** The efficiency of shTSP50 transfection was confirmed by western blot. β-actin served as an inner control. **b** BrdU assay showed a significant decrease in BrdU uptake in shTSP50 cell compared to that of the control groups. (** *P* < 0.01). **c** Cell count experiment detected remarkable reduction of cell number in shTSP50 cell compared to that of the control groups. (** *P* < 0.01). **d** In wound closure assay, the migratory speed of shTSP50 cell decreased significantly compared to that of the control groups. (** *P* < 0.01). **e** In the transwell assay, migrated cells significantly reduced after transfected with shTSP50. (** *P* < 0.01). In the transwell invasion assay, invasiveness was quantified by cells through Matigel and it was showed fewer cells in shTSP50 BGC-823 cell than control groups. (** *P* < 0.01). All data are expressed as the mean ± SD from three independent experiments. Scale bar = 100 μm

In addition to overexpression, we down-regulated TSP50 in gastric cancer cells in BGC-823 cell by its shRNA. The knockdown efficacy of TSP50 shRNA was confirmed by western blot analysis (Fig. 3a). As expected, BrdU incorporation and cell count assays indicated that cell proliferation was markedly suppressed by reducing TSP50 expression (Fig. 3b, c). Wound healing and transwell assay revealed that knockdown of TSP50 significantly inhibited migratory and invasive abilities of BGC-823 cell (Fig. 3d, e).

### TSP50 is negatively correlated with E-Cadherin in human gastric cancer

**Fig. 4:**
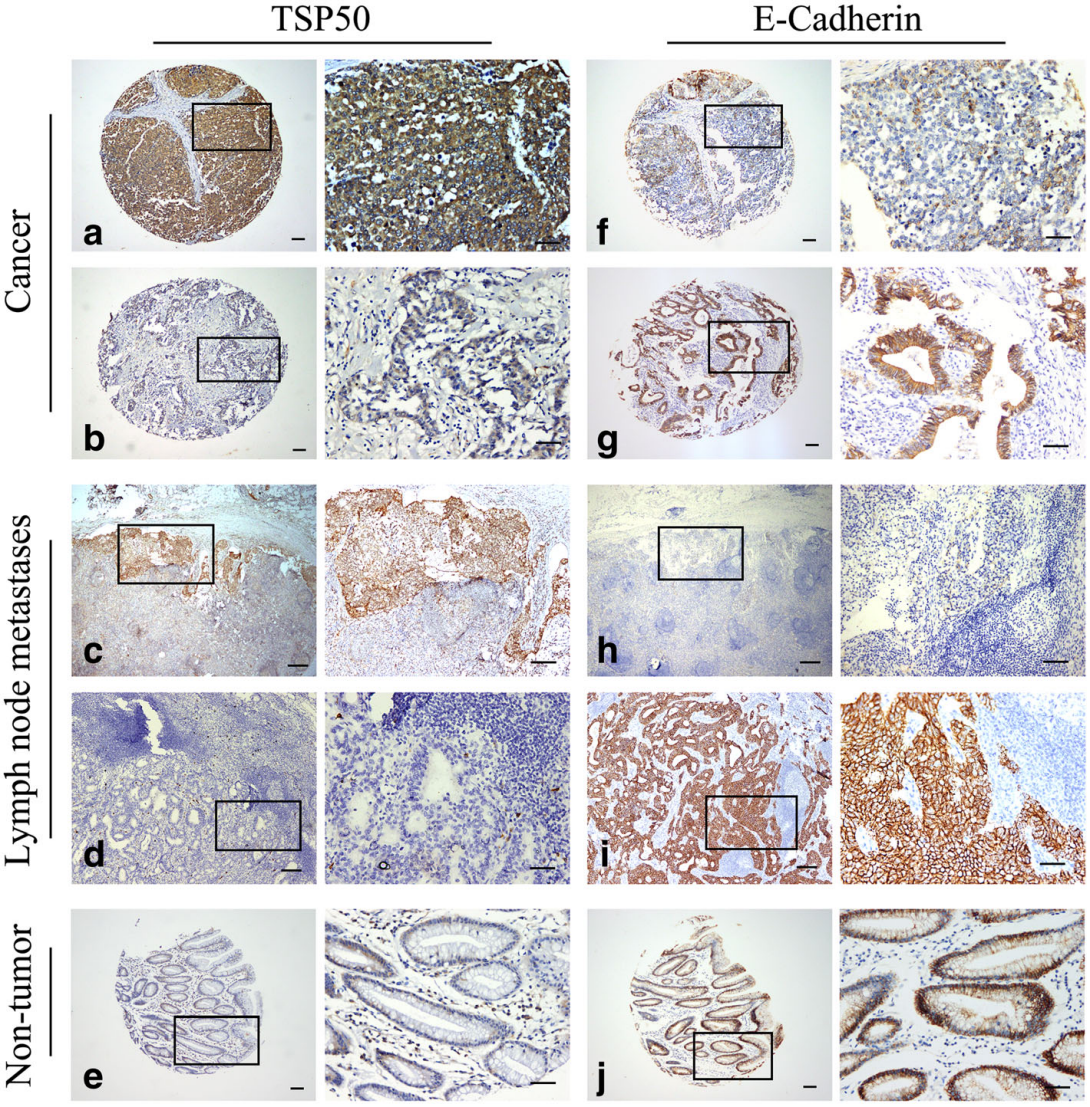
The negative correlation between TSP50 expression and E-Cadherin expression in gastric cancer tissues. **a, b, c, d** Representative images of high or low expression of TSP50 immunostaining in gastric cancer and lymph node metastasis lesion. **f, g, h, i** Immunostaining of E-Cadherin showed the contrary status in the same case compared with TSP50 immunostaining. **e** Low expression of TSP50 and (**j**) high expression of E-Cadherin in the same normal gastric mucosal tissue. In panel TSP50 and E-cadherin, the right panels displayed representative TSP50 and E-cadherin proteins expression in selected zone with enlarged view. Scale bar = 100 μm

E-Cadherin, a transmembrane glycoprotein, plays an important role in maintaining cell-cell adhesion. Loss of E-Cadherin is a hallmark of EMT and contributes to gastric cancer development [20–22]. Here, we tested the potential correlation between TSP50 and E-Cadherin expression in human gastric cancer tissues. The relationship between the expression of TSP50 and E-Cadherin was examined by immunohistochemistry (IHC) in tissue microarray containing 334 gastric cancer patients and 30 lymph node metastases. We found that in gastric cancer tissues which showed low expression of TSP50, 60.1% cases maintained high expression of E-Cadherin whereas in those which showed high expression of TSP50, only 37.2% cases remained to be high expression for E-Cadherin. Employing Phiand Cramers V correlation analysis, we found a significantly negative correlation between TSP50 and E-Cadherin expression (v = − 0.228, *P* = 0.000) not only in gastric cancer tissues, but also in lymph node metastases and adjacent non-tumor gastric mucosa (Fig. 4, Table 1, Additional file 1: Table S1).

**Table 1:** The relationship between TSP50 expression and E-Cadherin expression in gastric cancer tissues through Phi and Cramers V correlation analysis

Variables		All cases	E-Cadherin		*P* value	Phi
			Low(%)	High(%)		
TSP50	Low(%)	143	57(39.9%)	86(60.1%)	0.000	−0.228
	High(%)	191	120(62.8%)	71(37.2%)		

### Combination of TSP50 and E-Cadherin improves the prognostic stratification and prediction for lymph node metastasis in gastric cancer patients

**Fig. 5:**
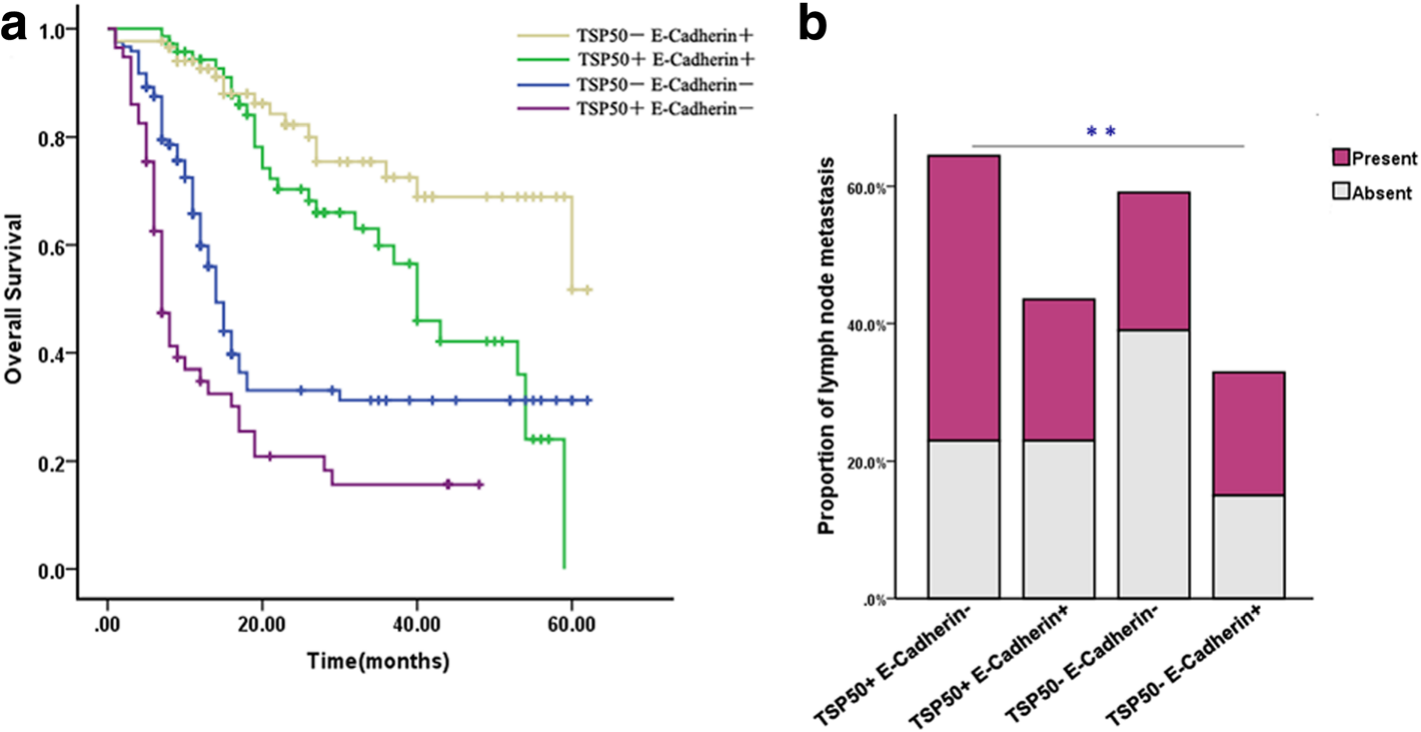
Combination of TSP50 and E-Cadherin improves prognostic value for gastric cancer patients, and is closely related to lymph node metastasis. **a** Kaplan–Meier estimated of overall survival of gastric cancer patients. Patient groups were separated according to expression of TSP50 and E-Cadherin. High expression of TSP50 and low level of E-Cadherin group had the worst overall survival rates, whereas low TSP50 expression and high E-Cadherin expression group had the best prognosis. **b** High expression of TSP50 and low level of E-Cadherin group was closely related to the present status of lymph node metastasis. (** *P* < 0.01)

Since either high expression of TSP50 or decreased E-Cadherin expression predicts a poor prognosis of gastric cancer patients [11, 23], and there was a negative relationship between them. Therefore, we analyzed whether the combination of TSP50 and E-Cadherin was a more powerful tool for prognostic prediction of gastric cancer. Based on the results from IHC, all 334 specimens were divided into four groups: high expression of TSP50 and low level of E-Cadherin (TSP50+ E-Cadherin-, *n* = 120), both high or low level of TSP50 and E-Cadherin (TSP50+ E-Cadherin+, *n* = 71 or TSP50- E-Cadherin-, *n* = 57), low expression of TSP50 and high level of E-Cadherin (TSP50- E-Cadherin+, *n* = 86). As showed in Fig. 5a, high TSP50 expression and low E-Cadherin expression group had the worst overall survival rates, whereas low TSP50 expression and high E-Cadherin expression group had the best prognosis. Moreover, high expression of TSP50 and low expression of E-Cadherin group was notably related to present lymph node metastasis (Fig. 5b).

### TSP50 induces EMT in gastric cancer cells

**Fig. 6:**
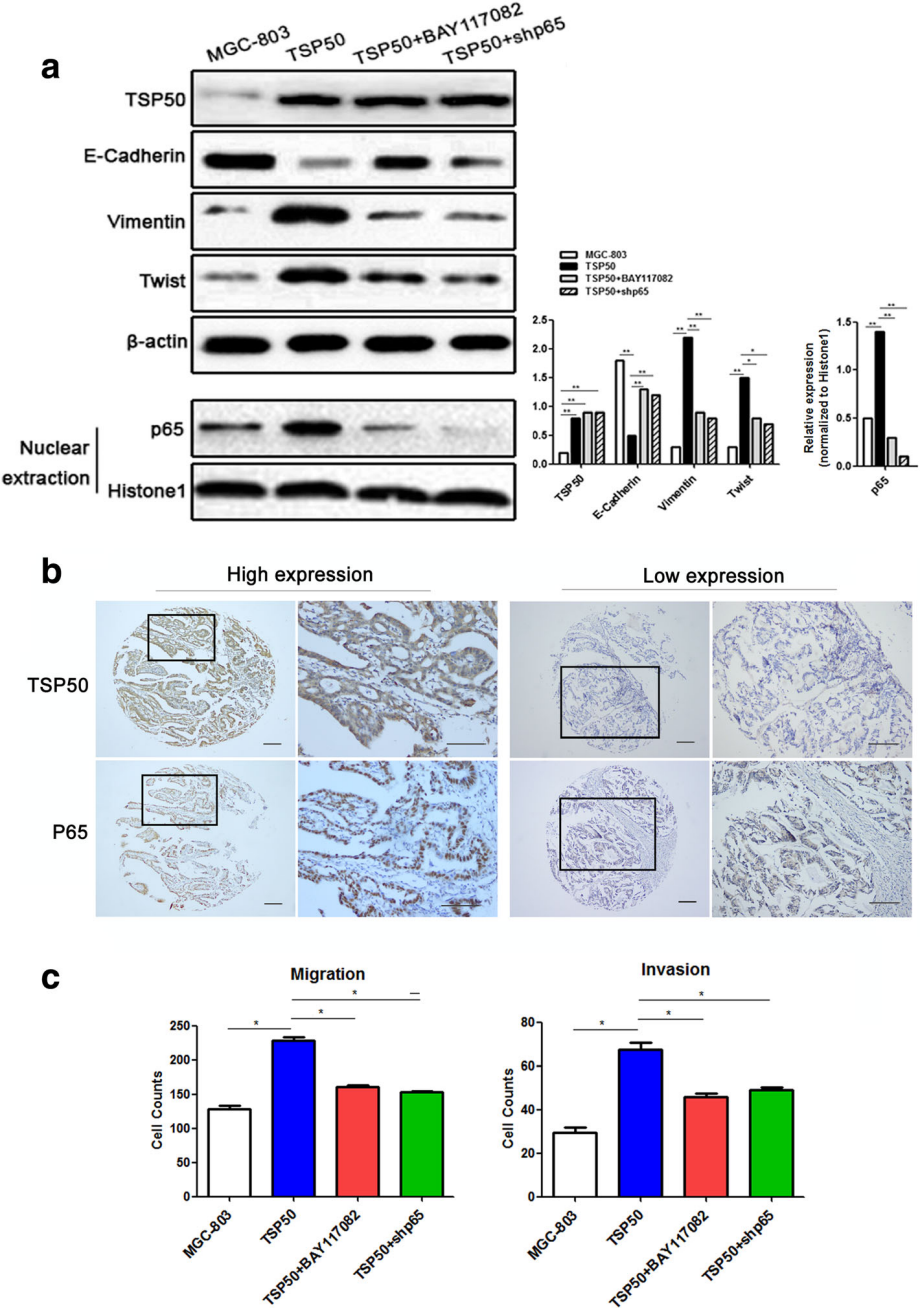
TSP50 induces EMT through NF-κB signaling pathway. **a** Expression levels of TSP50, E-Cadherin, Vimentin, Twist and nuclear p65 in MGC803 cell were determined by western blot analysis. β-actin and Histone1 were served as inner control of total protein and nuclear protein respectively. (*P < 0.05, **P < 0.01) (**b**)The positive association between TSP50 and p65 expression was showed in the same cohort of clinical gastric cancer tissue microarray using immunohistochemical staining. In panel high expression and low expression, the right panels displayed representative TSP50 and p65 proteins expression in selected zone with enlarged view. Scale bar = 100 μm. **c** Treatment with BAY117082 or transfection of shp65 reverted the effect of TSP50 on the migration and invasion in MGC803 cell partially. (*P < 0.05). All data are expressed as the mean ± SD from three independent experiments

The negative relationship between TSP50 and E-Cadherin was found in gastric cancer tissues and lymph node metastasis. It is known that down-regulation of E-cadherin expression is a significant feature of EMT [20–22]. Hence, it is of interest to detect the relationship between TSP50 and EMT in gastric cancer. We analyzed the protein levels of EMT markers including cell-surface protein E-Cadherin, cytoskeletal marker Vimentin, and transcription factor Twist in MGC-803 cell following transfection with TSP50 overexpressing plasmid. As shown in Fig. 6a, the expression of Vimentin and Twist was significantly increased, whereas the expression of E-Cadherin was decreased in MGC-803 cell stably transfected with TSP50 expression plasmid compared to control vector transfected cell. These results suggest that progression-promoting effect of TSP50 could be attributed to EMT induction in gastric cancer cells.

### NF-κB signaling pathway is involved in TSP50 induced EMT and invasion in gastric cancer cells

Based on the well-known fact that NF-κB signaling pathway has a crucial role in promoting EMT of tumor cells [12, 13, 16–18]. We then explored whether NF-**κ**B signaling pathway is involved in TSP50 induced EMT in gastric cancer cells through both pharmacological and genetic approaches. First, p65 nuclear accumulation, the hallmark of NF-κB signaling activation, was evaluated in MGC-803 cell following transfection with TSP50 overexpressing plasmid. Consistent with our hypothesis, expression of nuclear p65 was significantly elevated in TSP50 overexpressing MGC-803 cell when compared to control, however, treatment of BAY117082 or transfection with shp65 did not affect the level of TSP50 (Fig. 6a). Moreover, we evaluated the nuclear p65 level in the same cohort of gastric cancer tissues microarray using immunohistochemistry, the results showed that the expression of nuclear p65 was significantly positively related with TSP50 expression (Fig. 6b, Additional file 1: Table S2). These data suggested that TSP50 activates NF-κB signaling in GC cell. Then, we checked the expression of EMT related markers in TSP50 overexpressed cells after treated with BAY117082 or shp65, our results showed that inhibition of NF-κB signaling could notably down-regulate Vimentin and Twist, but up-regulate E-Cadherin (Fig. 6a). To further investigate whether NF-κB signaling participate in TSP50 induced cancer cell invasion, we detected the effect of inhibition of NF-κB signaling on migration and invasion of TSP50 overexpressed cancer cells. The results indicated that silencing of NF-κB signaling using BAY117082 or shp65 could partially revert the ability of invade and migrate in gastric cancer cell which were up-regulated by overexpression of TSP50 (Fig. 6c). These data demonstrated that NF-κB signaling pathway contributes to the effects of TSP50 on EMT phenotype and invasion of gastric cancer cells.

## Discussion

The present study has provided the first evidence concerning the role of TSP50 in gastric cancer. Our data showed that: (1) TSP50 was significantly up-regulated in most of the gastric cancer cell lines, and contributed to their proliferation and invasion; (2) TSP50 was negatively related with E-Cadherin expression in gastric cancer tissues as well as lymph node metastasis, and combination of TSP50 and E-Cadherin improved the prediction for prognosis and lymph node metastasis; (3) overexpression of TSP50 induced EMT through activating NF-κB signaling pathway to promote gastric cancer metastasis.

TSP50 was first identified in human breast cancer. After that TSP50 was shown to be involved in proliferation and metastasis of various cancer cells. In P19 murine embryonal carcinoma stem cells, knockdown of TSP50 inhibited cell proliferation and induced apoptosis [24]. Similar results were illustrated in laryngocarcinoma and cervical cancer [9, 10]. Song et al. recently found that TSP50 overexpression facilitated breast cancer cells motility and contribute to the development of metastasis both in vitro and in vivo [15]. These data suggested that TSP50 may serve as a common mechanism to promote tumorigenesis in different types of cancers. Consistently, we found that TSP50 was elevated in most of the gastric cancer cell lines, and overexpression or knockdown of TSP50 significantly affected cellular proliferation, migration and invasion.

EMT, characterized by loss of epithelial features (e.g. E-Cadherin) and acquired characteristics of mesenchymal cells (e.g. Vimentine, Twist), facilitate cells motility and invasion during the tumor development [18, 25]. Accumulating evidence has established the role of aberrant EMT activation in gastric tumorigenesis and cancer progression. However, the mechanism by which EMT is activated in the carcinogenesis of gastric cancer remains elusive [26, 27]. Recent study showed that TSP50 promotes cell invasion and metastasis by augmenting matrix metalloproteinase-9 expression in human breast cancer [15]. Our results showing modulation of TSP50 altered the phenotypes of gastric cancer cells in vitro prompted us to investigate whether EMT is the primary downstream target of TSP50 regulated effects. Since loss of epithelial marker E-Cadherin is one of the most important molecular events during EMT [20–22], we examined the expression pattern of E-Cadherin and its relation with TSP50 level in tissue microarray of a large number of archived human gastric cancers. First, we found that TSP50 overexpression was significantly correlated with E-Cadherin down-regulation in primary gastric cancer tissues and lymph node metastasis, and combination of them was a more powerful predictor for gastric cancer prognosis. Further, we showed that overexpression of TSP50 up-regulated mesenchymal maker Vimentin, EMT related transcript factor Twist and down-regulated epithelial marker E-Cadherinin in gastric cancer cells. Therefore, these data support the fact that TSP50 acts by enhancing EMT in gastric cancer progression. In fact, several oncogenic CTAs were recently shown to be involved in EMT and cancer metastasis. In this regard, CT45A1 is a potent inducer of the expression of the EMT master gene Twist1 in breast cancer and thereby promotes tumor invasion, and metastasis [28]. SSX (CTA5) was reported to be interacted with β-catenin leading to alterations of the transcription profile of target genes including Snail-2, E-cadherin, and Vimentin [29]. In gastric cancer, there were a few studies showing an association between CTAs and poor prognosis [30, 31]. However, the mechanism underlying its pathogenesis is rather lacking. Our study thus provided the first mechanistic evidence corroborating the important role of TSP50 in promotion of EMT and metastasis in gastric cancer.

Our study also demonstrated that TSP50 activated EMT in a NF-κB dependent manner in gastric cancer cells. NF-κB signaling plays a critical role in promoting and maintaining invasiveness of cancer cells via controlling of EMT process in different tumors including gastric cancer [16–18, 32, 33]. An earlier study also showed that NF-kB is required in TSP50-induced migration and invasion of breast cancer cells [15]. However, the effect of TSP50 on NF-kB in gastric cancer has not been reported in the literature. Therefore, our data provide a critical link between TSP50 and NF-kB in terms of gastric cancer progression. Nevertheless, given the fact that NF-kB signaling is broadly involved in the regulation of metastasis [17, 34–36], other signaling pathway other than EMT may also account for the TSP50 dependent invasive phenotype in gastric cancer. For instance, NF-κB activation was required for the transcription of a group of adhesion molecules including endothelial-leukocyte adhesion molecule-1 (ELAM-1) and intercellular adhesion molecule-1 (ICAM-1), which facilitate the extravasation of cancer cells [37, 38]. NF-κB binding sites were identified in the promoters of genes that encode several matrix metalloproteinases (MMPs) including MMP-2, MMP-9, and so forth, which degrade the extracellular matrix (ECM) to facilitate tumor cell invasion in tissues [39]. Further studies are needed to address these concerns in the pathogenesis of gastric cancer progression. Furthermore, it should be noted that inhibition of NF-κB signaling activity by its specific inhibitor BAY117082 or shp65 did not completely abrogate the TSP50 mediated activation of EMT, migration and invasion of gastric cancer cells, suggesting that other molecular mechanisms might be involved. Various cell signaling pathways are involved in the regulation of EMT, including Wnt/β-catenin, TGF-β, Notch, Hedgehog and others [40–43]. The pathways involved in TSP50-induced EMT besides NF-κB are also needed to be investigated in our future studies.

## Conclusions

In summary, this study delineates the pathophysiological role of TSP50 in gastric cancer progression. We showed that TSP50 underlies the pathogenesis of EMT, invasion and metastasis of gastric cancer by activating NF-κB signaling. TSP50 may prove to be clinically useful for developing novel therapeutic strategy for gastric cancer.

## Additional file

Additional file 1: Table S1. The relationship between TSP50 expression and E-Cadherin expression in lymph node metastatic lesions through Phi and Cramers V correlation analysis. **Table S2.** The relationship between TSP50 expression and nuclear p65 expression in gastric cancer tissues through Phi and Cramers V correlation analysis. (DOCX 14 kb)

## Abbreviations

CTAs: Cancer/testis antigens
EMT: Epithelial-to–mesechymal transition
IHC: Immunohistochemistry
PCR: Polymerase chain reaction
TSP50: Testes-specific protease 50
