# Supplementary material for: Testes-specific protease 50 (TSP50) promotes invasion and metastasis by inducing EMT in gastric cancer
Source: BMC Cancer. 2018 Jan 23;18:94. doi: 10.1186/s12885-018-4000-y (PMC5781268; doi:10.1186/s12885-018-4000-y)
Supplement: Additional file 1: Table S1. — The relationship between TSP50 expression and E-Cadherin expression in lymph node metastatic lesions through Phi and Cramers V correlation analysis. Table S2. The relationship between TSP50 expression and nuclear p65 expression in gastric cancer tissues through Phi and Cramers V correlation analysis. (DOCX 14 kb) [file 12885_2018_4000_MOESM1_ESM.docx]

| **Variables** | | **All cases** | **E-Cadherin** | | ***P* value** | **Phi** |
| --- | --- | --- | --- | --- | --- | --- |
|  |  |  | Low(%) | High(%) |  |  |
| **TSP50** | Low(%) | 13 | 5(38.5%) | 8 (61.5%) | 0.008 | -0.473 |
|  | High(%) | 17 | 12 (70.6%) | 5(39.4%) |  |  |

**Additional file 1**

**Table S1** The relationship between TSP50 expression and E-Cadherin expression in lymph node metastatic lesions through Phi and Cramers V correlation analysis

**Table S2** The relationship between TSP50 expression and nuclear p65 expression in gastric cancer tissues through Phi and Cramers V correlation analysis

| **Variables** | | **All cases** | **p65 (nuclear staining)** | | ***P* value** | **Phi** |
| --- | --- | --- | --- | --- | --- | --- |
|  |  |  | Low(%) | High(%) |  |  |
| **TSP50** | Low(%) | 143 | 49(34.3%) | 94(65.7%) | 0.001 | 0.347 |
|  | High(%) | 191 | 68(35.6%) | 123(64.4%) |  |  |
